# Cognitive Reserve: White Matter, Microvascular, Glymphatic and Cholinergic Dysfunction in Alzheimer’s Disease

**DOI:** 10.3390/life16101684

**Published:** 2026-10-09

**Authors:** Paul L. Wood

**Affiliations:** Metabolomics Unit, College of Veterinary Medicine, Lincoln Memorial University, 6965 Cumberland Gap Pkwy., Harrogate, TN 37752, USA; paul.wood@lmunet.edu

**Keywords:** Late-onset Alzheimer’s disease (LOAD), late-myelinating tracts, white matter dysfunction, retrogenesis, glymphatic system, cholinergic hypothesis, microvascular hypothesis, N-acyl-phosphatidylserines (NAPS)

## Abstract

Late-onset Alzheimer’s disease (LOAD) is a cognitive disorder that leads to the requirement of full-time care. The ever-increasing growth of the LOAD population demands that more research be dedicated to the discovery/design of effective therapeutics. The two major hypotheses that have been evaluated in the clinic include the cholinergic and the amyloid hypotheses. Unfortunately, the therapeutics that have resulted from research in these areas have minimal clinical impact. These are, respectively, the cholinesterase inhibitors and the anti-amyloid therapies. Clearly, a new path forward is needed. To this end, a number of genetic approaches and biochemical investigations are currently being employed to provide new insights into the pathophysiology of dementia. While there currently is a diverse array of dysfunctions that have been reported in LOAD subjects, white matter (WM) dysfunction may be a nodal point that is critical to the pathophysiology of cognitive decline. In this review, I present our current knowledge of WM dysfunction in LOAD. This addresses the potential contributions of microvascular dysfunction to WM abnormalities and neuronal dysfunction. This knowledge base has the potential to lead to a new approach for treating cholinergic dysfunction in cognitive disorders.

## 1. Introduction

Late-onset Alzheimer’s disease (LOAD) is characterized by a cascade of biological changes that potentially contribute to the pathophysiology of cognitive dysfunction after the age of 65. Defining the contributions of individual factors has not been successful to date. While there have been extensive efforts to identify genetic risk factors for LOAD, apolipoprotein E4 (ApoE4) remains the strongest genetic risk factor for LOAD. ApoE4 is involved in the regulation of lipid metabolism and also is a risk factor for microvascular pathologies in deep WM [1,2,3,4]. WM dysfunction, as reflected by WM hyperintensities (WMH) in imaging studies, is presumed to be reflective of these microvascular pathologies but also may involve non-vascular mechanisms [5]. Whole genome deconvolution studies have reported potential epigenetic biomarkers of resistance to the development of LOAD [6]. This may represent a new avenue of research capable of defining genetic factors critical to the pathophysiology of dementia. In this review, I attempt to relate these WM issues to the recognized cholinergic dysfunction in LOAD and how this may lead to new therapeutic approaches.

## 2. Retrogenesis: Late-Myelinating Tracts in LOAD

Imaging of WM hyperintensities (WMH) has generated enormous amounts of data supporting white matter dysfunction in LOAD [3,5,7,8,9,10,11,12]. This solid foundation led to the study of a large subject population comprising 3213 cognitively unimpaired, 972 with mild cognitive impairment (MCI), and 282 with LOAD [13] subjects, supporting a possible correlation of WM dysfunction with cognitive decline.

**Figure 1:**
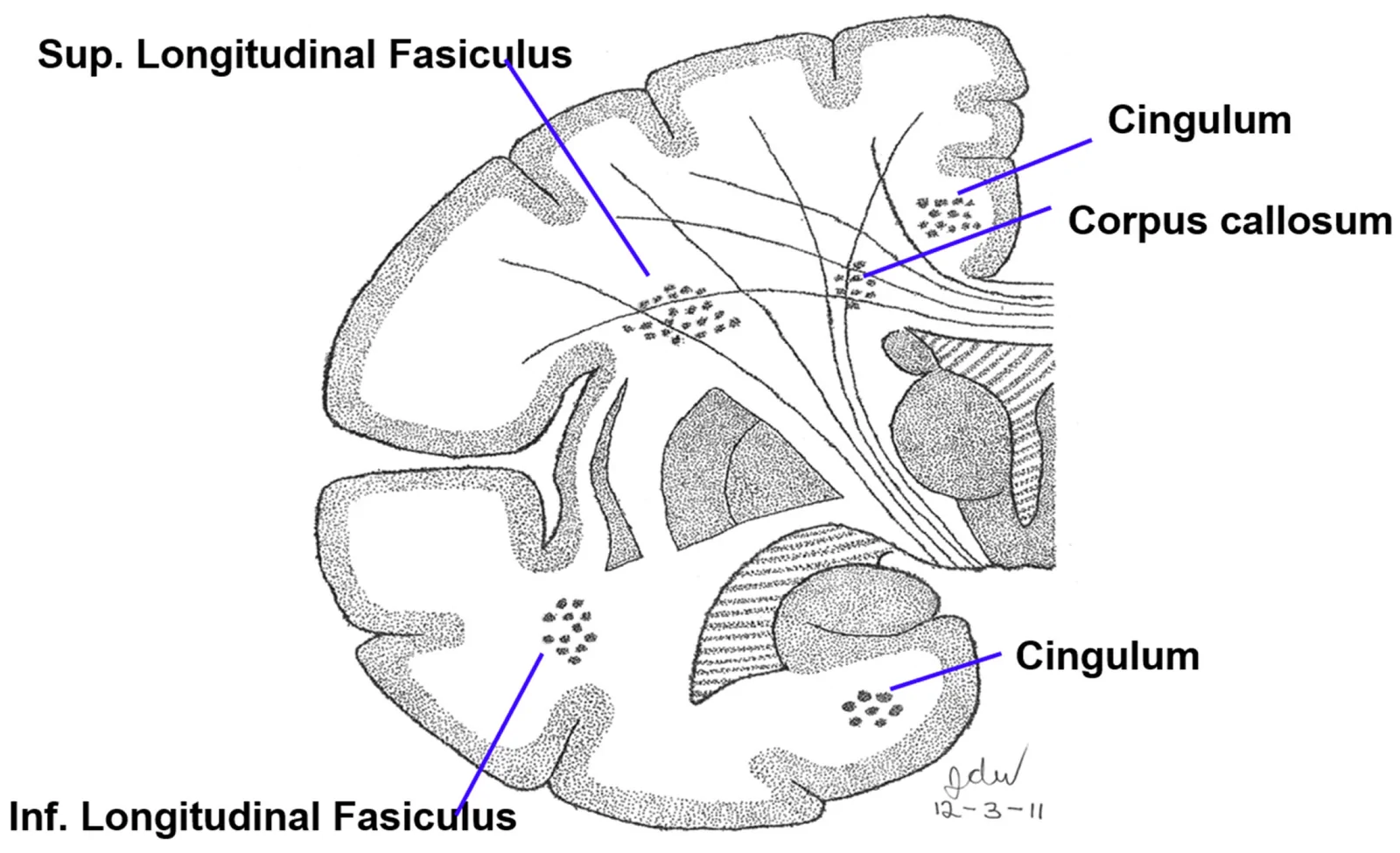
An anterior brain cross-section depicting the paths of late-myelinating association fiber tracts. The superior longitudinal fasciculus (SLF) is a longitudinal tract connecting the parietal and frontal lobes. The inferior longitudinal fasciculus (ILF) connects the occipital and anterior temporal cortices. The cingulum connects the cingulate and entorhinal cortices.

These imaging defects were found to be most pronounced in developmentally late-myelinating tracts (Figure 1) and correlated with semantic verbal fluency in LOAD. These include the superior [14,15,16,17] and inferior [14,18,19,20] longitudinal fasciculi, cingulum [13,21,22] and late-myelinating tracts within the fornix [16]. Since these imaging abnormalities appear first in late-myelinating tracts, this has been coined retrogenesis [22], in that WM dysfunction recapitulates aspects of developmental myelination in reverse order.

**Figure 2:**
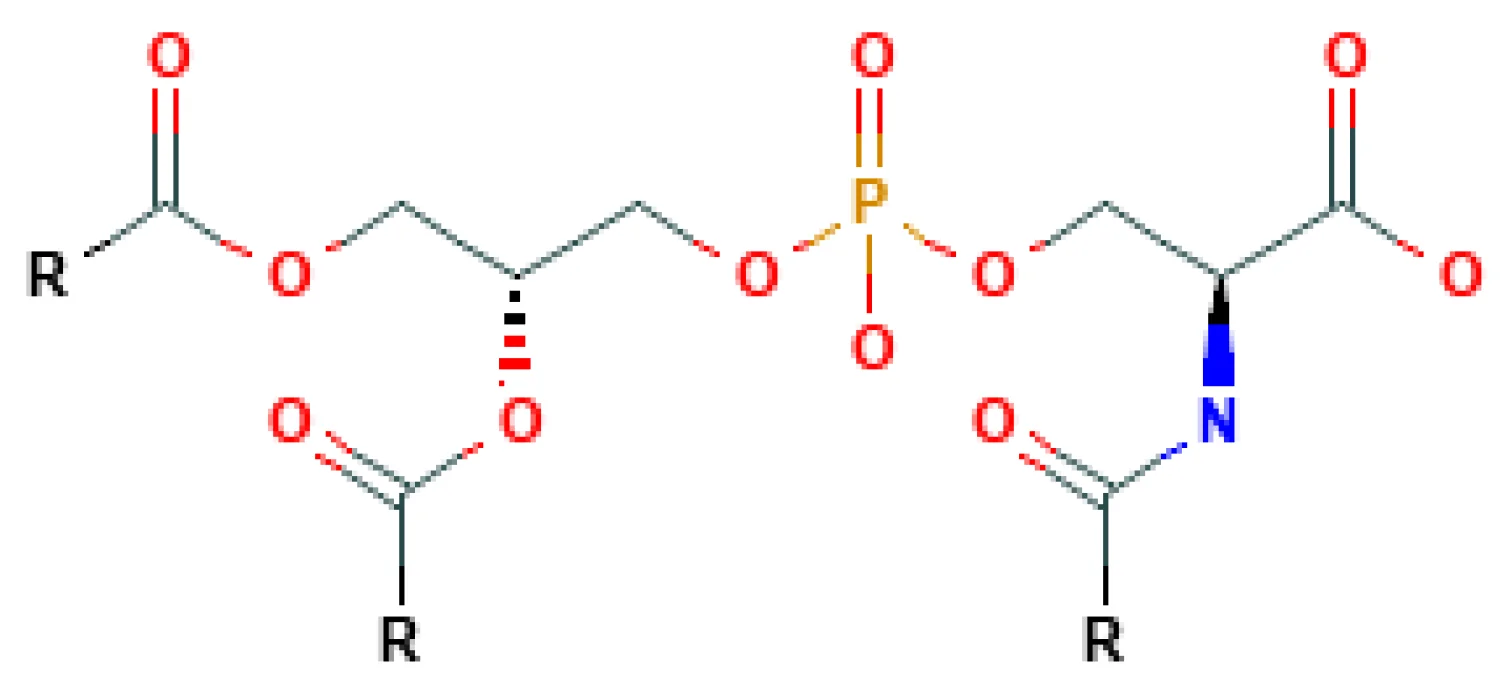
Core structure of NAPS. The base molecule is a phosphatidylserine with acyl fatty acids on the glycerol backbone. The major modification to this membrane lipid is N-acylation of the phosphoserine headgroup.

At the biochemical level, there have been limited lipid alterations found in LOAD WM [23]. However, more recent studies have reported small decreases in serine ether glycerophospholipids [24] and large decreases (approximately 50%) in N-acylphosphatidylserines [25] in WM in LOAD. These data suggest that N-acylphosphatidylserines (NAPS) may play an important role in the membrane structure of oligodendrocytes and that precursor molecules may be an experimental approach to be investigated. This large decrease in WM NAPS (Figure 2) in LOAD represents a potential lipid biomarker of WM dysfunction and should stimulate research to increase our understanding of the role(s) of NAPS in oligodendrocyte function.

## 3. Vascular Dysfunction/Hypometabolism in LOAD

Decreased cerebral perfusion is possibly the earliest dysfunction [26] monitored in LOAD patients utilizing diverse imaging approaches (perfusion PET, SPECT, and MRI). Monitoring perfusion of WM vasculature is the most complicated [27] but Arterial Spin Labeling is a non-invasive MRI technique that has measured blood flow in periventricular and subcortical WM [28]. Vascular function in LOAD also is more complicated to assess in that the incidence of microbleeds is double that of a comparatively aged control patient group [29]. In this regard, WMH (see Section 2) volumes appear to be linked to small vessel dysfunction early in the process of LOAD [30]. Decreased cortical hypometabolism in LOAD, as monitored routinely with [^18^F]fluorodeoxyglucose-positron emission tomography [31], is presumably a consequence of decreased cerebral perfusion. Altered blood supply and decreased energy sources in WM could have a strong negative consequence on both the cell bodies and nerve terminals associated with those affected WM tracts.

Research efforts to develop more cost-effective evaluation of vascular damage in the elderly have demonstrated that retinal microvasculature offers a non-invasive and sensitive approach that can detect microvascular damage in LOAD but not cognitively unimpaired individuals [32]. Longitudinal studies have further demonstrated that these alterations can be detected in MCI patients [33].

## 4. Glymphatic Dysfunction in LOAD

The brain’s glymphatic system is a paravascular network that conducts cerebrospinal fluid flow into the interstitial space with subsequent passage into meningeal and cervical lymphatic drainage vessels [34]. This network facilitates the circulation of cerebrospinal and interstitial fluids, which remove metabolic waste products. MRI has demonstrated decreased glymphatic function in LOAD subjects; however, debate over the reliability of current methods exists [35]. This lymphoid system also regulates brain water levels with aquaporin-4 (AQ-4), the main water transport channel in the CNS, present on astrocytic end feet, which form the perivascular spaces with microvessels. Since perivascular AQ-4 levels are decreased in LOAD [36], a number of investigators have hypothesized that perivascular dysfunction contributes to the pathogenesis of LOAD [37,38,39] leading to the suggestion that AQ-4 represents a tenable new target for treating LOAD [40,41].

## 5. Cholinergic Dysfunction in LOAD

The major cholinergic pathways involved in memory function have high neurotransmitter turnover rates as determined by precursor labeling methods, in the septal-hippocampal [42,43] and the nucleus basalis of Meynert (nbM)-cortical [43,44] cholinergic pathways. The major clinical evidence of the roles of these pathways in cognition results from studies of the cognitive deficits induced by treating subjects with centrally acting anticholinergics [45,46,47,48] and from decrements in choline acetyltransferase (ChAT) in both the cortical and hippocampal cholinergic afferents (Reviewed by Hampel et al., [47]). In MCI subjects, the septal-hippocampal cholinergic pathway also appears to make an attempt to adapt to axonal dysfunction by augmenting ChAT production in the hippocampus [49]. It is important to note that as reported for late-myelinating fiber tracts, imaging has also detected WM dysfunction in the fornix, which contains the cholinergic septal-hippocampal projection [13,14,50,51,52,53]. It remains to be defined if this WM dysfunction is of anterograde, retrograde or intrinsic origin.

Subsequent to reports of decreased ChAT in the nerve terminal regions of cholinergic projections [54,55], investigators turned to analysis of the cell body regions of these tracts. Initial studies reported extensive cholinergic neuronal loss in the nbM-cortical pathway [56]. However, these studies quantitated only magnocellular neurons in the nbM. Subsequent in-depth studies validated that there was neuronal cell death in the nbM, but only in the range of 20 to 40% and that there remained a large number of shrunken dysfunctional cholinergic neurons that were not dead [57,58,59,60]. This raises the possibility of pharmacological treatments to restore the health of these shrunken cholinergic neurons. In theory, this approach should be superior to cholinesterase inhibitors, which are limited to potentiating sick cholinergic neurons. Restoring or augmenting neuronal cell body function would be superior since ChAT is synthesized in neurons and transported to the nerve terminals by axonal transport mechanisms. These neuronal data also may indirectly provide support for the retrograde degeneration hypothesis, which suggests that the initial insult is at the level of cholinergic nerve terminals [61,62].

## 6. Cognitive Reserve: Non-Demented Alzheimer’s Neuropathology (NDAN) Elderly

At this stage of LOAD research, it is also critically important to increase the study of elderly individuals who are cognitively normal but, when autopsied, present with significant amyloid deposition [63,64,65,66,67,68,69,70]. Amyloid deposition has also been monitored in this elderly population with in vivo amyloid imaging [71]. This encompasses a population of approximately 20% of elderly (>65 years) normal subjects and has been coined as Non-Demented Alzheimer’s Neuropathology (NDAN) but in the literature these populations have also been coined as Non-Demented High Pathology (NDHP) and Alzheimer’s Disease Resilient (ADR).

Studies of NDAN subjects have led to speculation of cognitive reserve or resistance to AD pathology, which may be biochemical and/or anatomical [72,73,74]. It is important to stress that these are nondescript terms for a phenomenon (i.e., cognitive decline) which we do not understand but for which we are attempting to dissect mechanisms. In this regard, studies have demonstrated lower levels of cortical neuroinflammation in NDAN subjects than observed in LOAD subjects. For example, while significant elevation of cortical IL-6 levels has been monitored in LOAD [75,76], Il-6 levels are not elevated in NDAN subjects [75]. Also, microglial function, which is required for regulation of synaptic integrity, is maintained in NDAN subjects [77].

Of potential major importance (see Section 5) are studies which monitored ChAT immunoreactive fibers [78] and biochemical measurements of ChAT [79] in NDAN subjects. These investigations detected only 20 to 30% decrements in ChAT in brain areas with high amyloid load, contrasting with widespread ChAT losses in LOAD. This maintenance of cholinergic transmission may be a critical factor in sustaining cognition in the presence of pathological burden in NDAN subjects (see Section 5).

## 7. Tau Tangles

In addition to extracellular amyloid deposition in LOAD, neuronal tau tangle formation occurs and with the failure of the amyloid hypothesis, renewed efforts to address tau tangles has taken place since there are strong correlations of tau pathology with cognitive decline [80]. In this regard, the HS3ST1 gene which has been identified as a risk locus for LOAD encodes 3-O-sulfotransferase isoform 1 (3-OST-1) responsible for the levels of sulfated heparan sulfate in the cortex of LOAD subjects [81]. Heparan sulfate proteoglycans serve a number of structural functions in the nervous system including in basement membranes, secretory vesicles and membrane receptors [82]. Also, sulfated heparins have been shown to regulate tau cellular uptake [81]. These data indicate that 3-OST-1 may be a new therapeutic target to be investigated. However, it is worthy to note that, in addition to amyloid deposition, there is significant tau tangle formation [83] and elevated levels of sulfated heparin sulfate [81] in NDAN subjects.

Research in this area is further complicated by mixed pathologies such as AD pathology in Parkinson’s subjects possessing extensive Lewy bodies [84].

## 8. Summary

While cholinesterase inhibitors [85] and anti-amyloid therapies [86] provide some slowing of cognitive decline, if utilized early in the AD disease process, more effective agents are needed. This has resulted in an increased effort to address alternative hypotheses of the pathogenesis involved in cognitive dysfunction. There are no established therapeutic targets for LOAD clinical development at this time. However, the concept of established therapeutic targets is dangerous based on the limited clinical success of cholinesterase inhibitors and anti-amyloid therapies. This review provides some tentative options to investigate:

- Investigation of biochemical approaches to supporting myelin function.
- Exploration of mechanisms to support/restore healthy microvascular function.
- Design of aquaporin-4 modulators to address perivascular dysfunction.
- Identification and implementation of trophic modulators to restore shrunken cholinergic neurons. In light of the maintenance of cholinergic neurotransmission in NDAN subjects, this approach may be of critical importance.
- Evaluation of parameters in NDAN subjects to increase our understanding of cognitive reserve:
  ○ Investigation of WM integrity (e.g., WH hyperintensities);
  ○ Study of vascular amyloid load;
  ○ Assessment of retinal microvasculature;
  ○ Evaluation of glymphatic function;
  ○ Modulation of 3-O-sulfotransferase isoform 1.

The complexity of these potential approaches is that many of the observed abnormalities in LOAD may be interlinked [87,88], but the sequence of critical pathological events is not clear, making the definition of a pivotal point of intervention elusive.

## Data Availability

All data is included in the manuscript.
